# Facilitation or disengagement? Attention bias in facial affect processing after short-term violent video game exposure

**DOI:** 10.1371/journal.pone.0172940

**Published:** 2017-03-01

**Authors:** Yanling Liu, Haiying Lan, Zhaojun Teng, Cheng Guo, Dezhong Yao

**Affiliations:** 1 The Lab of Mental Health and Social Adaptation, Faculty of Psychology, Research Center of Mental Health Education, Southwest University, Chongqing, China; 2 Key Laboratory for Neuro Information of Ministry of Education, School of Life Science and Technology, Center for Information in Medicine, University of Electronic Science and Technology of China, Chengdu, China; Bournemouth University, UNITED KINGDOM

## Abstract

Previous research has been inconsistent on whether violent video games exert positive and/or negative effects on cognition. In particular, attentional bias in facial affect processing after violent video game exposure continues to be controversial. The aim of the present study was to investigate attentional bias in facial recognition after short term exposure to violent video games and to characterize the neural correlates of this effect. In order to accomplish this, participants were exposed to either neutral or violent video games for 25 min and then event-related potentials (ERPs) were recorded during two emotional search tasks. The first search task assessed attentional facilitation, in which participants were required to identify an emotional face from a crowd of neutral faces. In contrast, the second task measured disengagement, in which participants were required to identify a neutral face from a crowd of emotional faces. Our results found a significant presence of the ERP component, N2pc, during the facilitation task; however, no differences were observed between the two video game groups. This finding does not support a link between attentional facilitation and violent video game exposure. Comparatively, during the disengagement task, N2pc responses were not observed when participants viewed happy faces following violent video game exposure; however, a weak N2pc response was observed after neutral video game exposure. These results provided only inconsistent support for the disengagement hypothesis, suggesting that participants found it difficult to separate a neutral face from a crowd of emotional faces.

## Introduction

Research has remained inconsistent regarding the effect of violent video game exposure on cognition. For example, cross-correlational, experimental, and longitudinal studies have indicated a close association between exposure to violent video games and aggression, see two meta-analysis [1,2]. However, recent research has produced contradictory, negative, findings [3,4], reporting no link between violent video game exposure and aggressive behavior. Further, a recent reanalysis challenged some previous results with the same data [5]. This study aimed to investigate attentional bias in facial affect processing after short-term exposure to violent video games and to examine the neural correlates of such effects.

### Debating the effects of violent video games

In recent years, the General Aggression Model (GAM) has been used by researchers to explain the association between violent video game exposure and aggressive behavior [6]. The GAM incorporates three mechanisms (i.e., cognitive, emotional, and arousal) by which individuals modify their behavior under the influence of individual (e.g., personality) and contextual variables (e.g., violent video games). According to this model, attentional bias has been reported to appear after exposure to violent media [7, 8], and this has been purported to be the underlying cause of aggressive behavior.

A number of studies have identified negative effects (e.g., aggressive cognitive or behavior) associated with individual exposure to violent video games [6–8]. Specifically, these studies have demonstrated changes in aggressive cue processing and emotional face recognition after violent video game exposure, which reflect alterations in cognitive bias. Such studies suggest that cognitive bias toward aggressive cues after violent video game exposure is due to increased activation of brain regions associated with aggressive cognition or scripting. According to the GAM, increased activation in these brain regions likely underlies the aggressive behavior that has been suggested to be associated with violent video game exposure [5, 9].

However, some researchers argue that the GAM does not effectively explain the link between violent video games and aggression [10]. A common criticism of the GAM is that no clear mechanism exists by which cognition and affect give rise to a meaningful outcome (i.e., aggressive behavior). For example, violent media, which is one of the most consumed media forms, has become increasingly popular in recent years; however, violent crime is at a historical low [10]. If the GAM provides an adequate explanation for aggressive behavior, there should be increasing evidence of violent crime. In addition, a significant number of studies have failed to identify a clear link between violent video games and increased aggression [11, 12]. Critics argue that video game experiments often suffer from poorly matched video game controls, wherein games employed in the neutral condition significantly differ on a variety of variables (e.g. difficulty or frustration) than games employed in the violent condition [2]. Despite the inconsistent results obtained with regard to the effects of violent video games, the mechanisms underlying such effects on social behavior require further investigation.

### The study of attentional bias in violent video games

Attentional bias is a key issue in studies investigating the effects of violent video games, as early attentional processes exert crucial influence on later cognitive decisions. More specifically, two opposing mechanisms have been described with regard to the effects of violent video games on affect processing (i.e., facilitation vs. disengagement) [13]. Attentional facilitation causes negative emotional information to be prioritized by individuals exposed to violent video games. For example, previous studies have reported that angry faces are identified more rapidly following violent video game exposure [7], indicating that attentional facilitation is triggered by exposure to violent media[5]. Furthermore, studies of event related potentials (ERPs) provide additional support for this mechanism, with higher N1 and P300 activity following violent video game exposure [14, 15].

However, it is difficult for individuals to discard information related to negative affect, a process known as attentional disengagement failure [13]. Interestingly, this process has also been linked to violent video game exposure. For example, Zhen, Xie, Hu, and Zhang (2013) found that long-term exposure to violent video games results in difficulty disengaging from angry faces when a target stimulus is displayed for 500 ms in a dot probe paradigm [16]. ERP studies have suggested evidence of reduced P300 amplitudes when participants are required to process negative emotions or images after exposure to violent video games [17–19]. However, some recent fMRI studies have failed to find brain activity changes with either short or long term exposure to violent video games [20, 21].

### Overview of the present study

Given the inconsistency in results regarding attentional regulation after exposure to violent video games, the present study aimed to investigate two differing mechanisms of attentional bias after short-term exposure to violent video games, and to characterize their neural correlates using ERP analysis. N2pc is an ERP component that is closely associated with attentional bias during visual information processing [22–24]. Analysis of N2pc might be useful for exploring the effects of violent video game exposure, especially with regard to the attentional selection process for facial emotion perception. Further, N2pc provides a temporally accurate marker of attentional object selection that can be used to study the visual search component of facial affect processing. Enhanced negativity at posterior electrodes, which are positioned contralateral to the visual field, indicates that an individual is focusing on objects that appear among distractors in visual search displays. This component is typically observed 200–300 ms after stimulus onset, and is generated by the lateral extrastriate cortex and inferotemporal visual areas. Studies suggest that this component reflects the allocation of spatial attention to objects that match target-defining features [22, 25–31].

Two potential mechanisms exist to explain the role of N2pc in the visual search process, including the facilitation of target stimuli and ignoring task-irrelevant stimuli to promote target-specific processing [30]. When stimuli consist of all emotional faces, the visual search task is referred to as an emotional search task. Emotional search tasks have been used to investigate individual sensitivity to threatening and aversive stimuli compared to positive and/or neutral stimuli [32–34]. During emotional search tasks, individuals are required to view an array of nine schematic faces and decide whether a discrepant face is present in the array. Some studies have identified an “angry or fearful face advantage effect” during this task [33,35], while others have observed a “happy face advantage effect” [36,37]. However, despite inconsistent advantages associated with exposure to “angry” or “happy” faces, individuals consistently elicit significantly higher N2pc amplitudes during selective attentional processing. Previous research provides strong evidence for the role of N2pc in the location of covert, consciously directed attention [22]. Based on these effects, attentional capture might reflect larger N2pc amplitudes for the identification of angry or happy faces during the emotional search task.

In the present study, two tasks were designed to explore the role of N2pc in emotional face processing following short-term violent video game exposure. In the facilitation task, participants were required to identify an emotional face in a group of neutral faces as quickly as possible (task A), while in the disengagement task, participants were required to rapidly separate a neutral face from angry and/or happy faces (task B). According to previous studies, the effects of violent video game exposure can be visualized by a reduction in the happy face advantage [7,8] and desensitization [17–19]. In the present study, we predicted that higher N2pc amplitudes, or a more significant N2pc presence, would be observed in response to the presentation of angry faces during the facilitation task (task A) in the group of participants exposed to violent video games. On the other hand, we predicted that attentional focus on emotional faces, either angry or happy, would be associated with difficulty disengaging from these faces. If this were the case, then lower N2pc amplitudes, or no significant N2pc, would be elicited in response to emotional faces in the group exposed to violent video games during the disengagement task (task B).

## Methods

### Ethics statement

Approval of the study was granted by the Human Research Ethics Committee of the Southwest University of China (SWUC). In compliance with the principles of the Declaration of Helsinki, all participants provided written informed consent prior to the start of experimentation. Further, the individuals in this manuscript have given written informed consent to publish these case details.

### Participants

Forty-five right-handed student volunteers were recruited from SWUC (32 males, 13 females; mean age = 21.12 ± 2.03 years) for the present study. Upon completion of the study, participants were paid RMB 40. All participants had normal or corrected-to-normal vision, and none had recently taken medication or suffered from neurological deficits (e.g., language impairment).

The sample size in the violent video game group was set to 23 (15 males, eight females). The neutral group (i.e., the control group) originally featured an identical sample size of n = 23; however, one participant quit the test prior to formal experimentation. This sample size (23 per group) was determined prior to recruiting participants and was based on previous ERP studies, which usually feature groups of 20 participants.

### Materials

Six video games were selected for use in preliminary studies. In accordance with previous violent video game research [18, 19, 38, 39], three neutral video games (i.e., *Avoid Tetris*, *Mega World Smash*, and *Babel Running*) and three corresponding violent video games (i.e., *Left 4 Dead*, *Grand Theft Auto*, and *Prototype*) were included. Following completion of the games, participants were required to complete the Game Evaluation Questionnaire (GEQ) [38], which measures a number of variables, including the frustration, enjoyment, difficulty, lack of pauses, action, violence content, and graphics associated with each game (1 = low to 7 = high).

Preliminary game evaluations included 57 participants (52 males, five females) who were not part of the formal experiment. Each participant was required to complete two randomly selected video game evaluations (one neutral and one violent video game). Participants played one game for 3 min in a practice round to ensure that they understood the rules. Participants then played the game for 20 min, at which point the experimenter stopped the game and asked them to complete the GEQ. After a 3-min break, participants followed the same procedure to evaluate another game.

Subsequent results indicated that *Avoid Tetris* and *Left 4 Dead* were best matched to the neutral and violent video game categories, respectively (Table 1). With regard to violent content and graphics, a one-way ANOVA demonstrated significantly higher scores for the violent video game (*Left 4 Dead*) than the neutral video game (*Avoid Tetris*) (*F*_(1,55)_ = 12.66, and *F*_(1,55)_ = 12.62, respectively, *p* < .01). However, no other features had significant effects (all *F* < 2, *p* > .05). Therefore, these two games were used in the formal experiment.

**Table 1:** Mean ratings and one-way ANOVA for a neutral and violent video game.

Dimension	*Avoid Tetris*	*Left 4 Dead*	*F*_(1, 55)_	*p value*
Difficulty	4.20 (1.69)	3.40 (0.99)	1.35	0.20
Lack of pauses	5.00 (1.50)	4.00 (1.35)	1.72	0.18
Action	4.30 (1.42)	4.13 (1.46)	0.28	0.78
Frustration	3.90 (1.91)	3.33 (1.50)	0.83	0.41
Enjoyment	4.20 (0.63)	4.60 (1.06)	1.18	0.25
Violent content	1.50 (0.71)	5.70 (0.88)	12.66	< .01
Violent graphics	1.30 (0.48)	5.73 (1.03)	12.62	< .01

Two hundred and forty affective facial images were selected from the Chinese Facial Affective Picture System (CFAPS) [40, 41]. Using Photoshop software, each image was converted to black-and-white and standardized to a set brightness and size of 7 × 6 cm. Distinguishing features were also removed from all faces. Fifty-seven students then rated the pleasure and arousal of each image from 1 (low) to 7 (high) in the preliminary experiment.

Happy faces with scores in pleasure and arousal lower than 5, angry faces with pleasure scores higher than 2 and arousal scores lower than 3, and neutral faces with scores in pleasure and arousal lower than 3 were removed from the selection according to previous studies [42]. Thirty angry, 30 happy, and 60 neutral faces were used in the formal experiment. The proportion of female to male faces was 1:1. One way-ANOVA results for each type of face are displayed in Table 2. A significant difference was observed with regard to arousal (*F*_(2, 110)_ = 515.24, *p* < .01) and pleasure (*F*_(2, 110)_ = 732.54, *p* < .01).

**Table 2:** Means and SD for the three kinds of facial emotion.

	Angry face	Neutral face	Happy face
Arousal	6.03 (0.61)	3.37 (0.58)	6.12 (0.53)
Pleasure	1.67 (0.38)	3.41 (1.03)	6.33 (0.45)

### Experimental tasks

**Fig 1:**
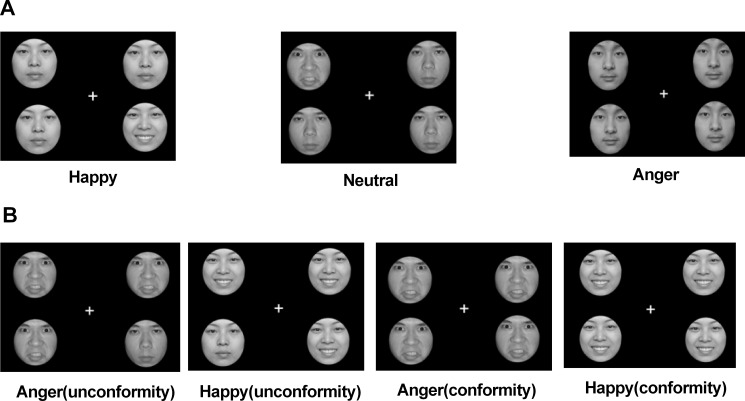
Emotional search task. (A) Representative images from the attentional facilitation task. (B) Representative images from the attentional disengagement task.

Emotional search task A was used to explore attentional facilitation. Participants were required to identify an emotional face among three other faces as quickly as possible. Three types of images were used for this task: a happy face with three neutral faces, an angry face with three neutral faces, and four neutral faces (**Fig 1A**).

Emotional search task B was used to examine attentional disengagement. In this task, participants were required to identify neutral faces among other facial stimuli as quickly as possible. Four types of images were used for this task, including a neutral face and three happy faces, a neutral face with three angry faces, four happy faces, or four angry faces (**Fig 1B**).

### Procedures

Subjects were asked to participate in an EEG/ERP experiment to assess the effects of video games on reaction speed. After participants were fitted with the EEG helmet and electrode jelly had been applied, they were randomly assigned to either the violent or neutral video game group. Participants then played their allocated game for 25 min and, following a 2-min break, 5-min training trials for the emotional search task were initiated.

The formal experiment began once as participants directed their gaze to a “+” fixation point on the screen for 200 ms. Participants were then shown four faces for 500 ms, and were asked to study the conformity of these four faces for 1800 ms, pressing “F” to indicate conformity and “J” to indicate nonconformity. A black screen was then displayed for 750–1050 ms. Tasks A and B contained 360 trials, and participants were given a 3-minute break every 90 trials. The location of the emotional faces was randomized. To control for order, half of the participants were allotted to task A then task B, while the other half were assigned task B and then A. All tasks lasted 40 minutes. Reaction time (RT) and accuracy were assessed for both tasks A and B.

### EEG recording and data analysis

Continuous EEG recordings were obtained using 64 Ag-AgCl unipolar leads on a 64-lead connection, which was consistent with the extended 10–20 system, and digitized at a sampling rate of 500 Hz (Brain Products GmbH). The impedance for all electrodes was maintained below 5 kΩ. The prefrontal electrode, at the midpoint between FPz and Fz (i.e., AFz), was connected to the ground using the frontal vertex (i.e., FCz) as the online reference. Participants were asked to refrain from blinking, to remain as still as possible, and to relax their facial muscles. Participants were then required to direct their gaze towards a fixation point presented on the screen. To control for eye movement artifacts, horizontal and vertical electrooculograms were recorded from electrodes positioned above and at the outer canthus of the right eye, respectively.

Offline analysis of EEG data was performed using Analyzer 2.0 software (Brain Products GmbH), with recordings re-referenced to the “infinity” reference provided by the reference electrode standardization technique (REST) [43]. Regression was performed to remove horizontal and vertical electrooculograms from the Analyzer 2.0 system. With a 0.01–30 Hz band pass filter and artifact rejection, this method was successfully used to eliminate artifacts with amplitudes exceeding ± 100 μV.

**Fig 2:**
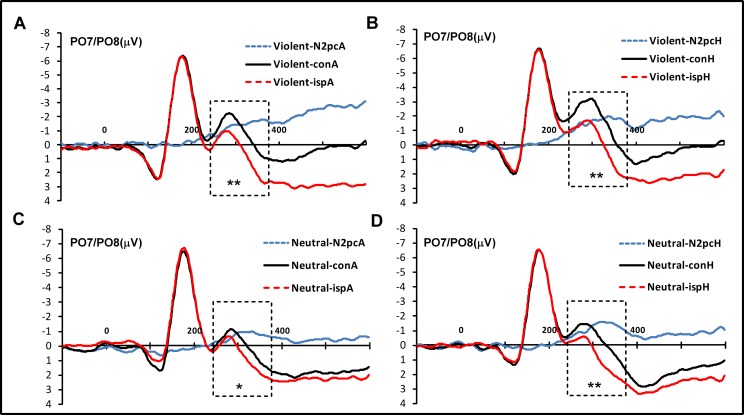
The N2pc for task A. (A) It represent the grand averages of N2pc in the violent video game group in response to angry faces. (B) It represent the grand averages of N2pc in the violent video game group in response to happy faces. (C) It represent the grand averages of N2pc in the neutral video game group in response to angry faces. (D) It represent the grand averages of N2pc in the neutral video game group in response to happy faces. *p < .05, **p < .01.

**Fig 3:**
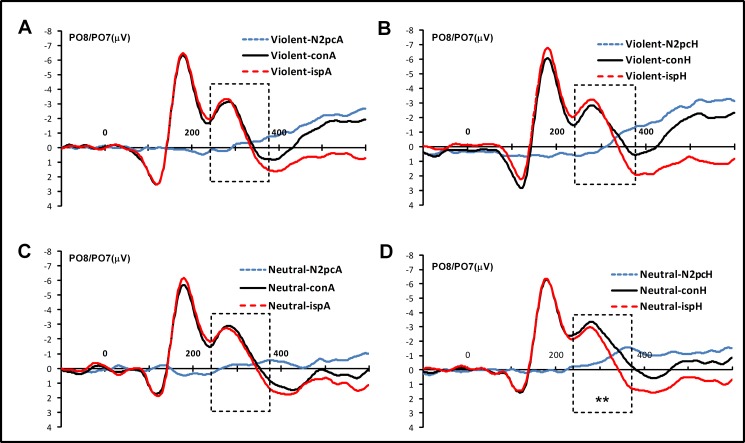
The N2pc for task B. (A) It represent the grand averages of N2pc in the violent video game group in response to angry faces. (B) It represent the grand averages of N2pc in the violent video game group in response to happy faces. (C) It represent the grand averages of N2pc in the neutral video game group in response to angry faces. (D) It represent the grand averages of N2pc in the neutral video game group in response to happy faces. *p < .05, **p < .01.

N2pc components were quantified based on ERP waveforms measured at the lateral posterior electrodes PO7 and PO8 [25,27]. An epoch of 700 ms and a baseline of 100 ms were established prior to stimulus onset. Ipsilateral and contralateral ERPs were computed for exposure to angry and happy faces. The time window for the N2pc component was 240–380 ms after stimulus onset [44]. The grand average of the N2pc for PO7/PO8 was calculated in different situations for task A (**Fig 2**) and task B (**Fig 3**). For RT and accuracy in task A, a repeated-measures ANOVA was conducted for two game types (violent and neutral) × three target faces (neutral, angry, and happy). For RT and ACC in task B, a repeated-measures ANOVA was conducted for two game types (violent and neutral) × two target faces (angry and happy) × two types of consistency (conformity and unconformity). For N2pc average amplitudes, a repeated-measures ANOVA was conducted for two game types (violent and neutral) × two target faces (angry and happy). N2pc was computed from contralateral minus ipsilateral ERP waveforms in response to angry and happy target faces. To confirm that N2pc components were reliably elicited in response to angry or happy faces, N2pc effects were compared with zero in two-tailed single-sample *t* tests. To measure effect sizes, Cohen’s d and partial eta-squared (*η*^2^_p_) values [45] were computed for all significant *t* or *F* tests comparing behavioral data and N2pc amplitudes.

For getting more reliable results in N2pc, game types and target faces were also analyzed using a Bayesian version of the repeated-measures ANOVA [46]. Bayes factors were calculated for the full model of the two-way interaction and for the main effect of game type and target faces, a model with a main effect but without an interaction, and a null model. *BF*_1,0_, which has been suggested to support the alternative hypothesis, is comprised of the following conventional cut-offs: A Bayes factor > 3 or < 1/3 represents substantial evidence; conversely, anything between 1/3 and 3 represents only weak or anecdotal evidence [47].

## Results

### Behavioral results

For task A, data was only available for 44 of the 45 participants, as one participant exhibited low accuracy (less than .50). Table 3 displays the behavioral statistics for task A. A two (game type: violent or neutral) × three (emotion: angry, happy, or neutral) repeated-measures ANOVA was performed. No significant main effects were detected for game type, emotion, or their interaction with regard to accuracy (all *F* < 2.50, *p* >.10). However, a significant main effect was detected for emotion (*F*_(2, 82)_ = 39.69, *p* < .01, *η*^2^_p_ = .49, *BF*_1,0_ = 9.54×10^9^) on RT, with shorter RTs in response to emotional faces (anger, *M* = 422.37, *SE* = 16.39; happy, *M* = 412.01, *SE* = 15.12) relative to neutral faces (*M* = 498.31, *SE* = 22.98). No difference in RT was observed between angry and happy faces (*p* > .05). In addition, no main effect was detected for game type and no interaction was identified between game type × emotion with regard to RT (all *F* < 2.41, *p* > .13).

**Table 3:** Accuracy and RT for task A (Mean, SD).

	Violent video game			Neutral video game		
	Angry	Happy	Neutral	Angry	Happy	Neutral
Accuracy	.85 (.11)	.88 (.08)	.85 (.10)	.86 (.08)	.89 (.09)	.88 (.09)
RT	399.88 (87.35)	390.16 (87.60)	463.18 (122.56)	444.86 (126.57)	433.86 (111.63)	533.43 (177.37)

For task B, data was available for 44 participants, due to low accuracy in one participant (less than .50); this was not the same subject that exhibited low accuracy in task A. Table 4 displays statistics for the behavioral results of task B. A repeated-measures ANOVA was conducted with regard to two game types (violent and neutral) × two target face (angry and happy) × two types of consistency (conformity and unconformity). For accuracy, main effects were observed with regard to consistency and target face (*F*_(1, 42)_ = 79.66, *p* < .01, *η*^2^_p_ = .65, *BF*_1,0_ = 9.69×10^36^; *F*_(1, 42)_ = 38.90, *p* < .01, *η*^2^_p_ = .57, *BF*_1,0_ = 6.58×10^20^, respectively). Higher accuracy was identified in conditions of conformity vs. unconformity (*M* = .92, *SE* = .01 vs. *M* = .80, *SE* = .01), and higher accuracy was identified when participants were presented with happy faces over neutral faces (*M* = .88, *SE* = .01, vs. *M* = .84, *SE* = .01). No main effect was found for game type (*F* <1.00, *p* > .20, *η*^2^_p_ = .01, *BF*_1,0_ = .96). However, an interaction of emotion × consistency was identified (*F*_(1, 42)_ = 9.49, *p* < .01, *η*^2^_p_ = .18, *BF*_1,0_ = 7.65). Simple effect tests indicated higher accuracy in unconformity than conformity for angry faces compared with happy faces. No other two- three-way interactions were detected for accuracy (all *F* < 1.00, *p* > .20).

**Table 4:** Accuracy and RT for task B (Mean, SD).

	Violent video game				Neutral video game			
	Conformity		Unconformity		Conformity		Unconformity	
	Happy	Anger	Happy	Anger	Happy	Anger	Happy	Anger
Accuracy	.94 (.06)	.91 (.07)	.82 (.09)	.77 (.08)	.93 (.06)	.91 (.06)	.83 (.07)	.77 (.08)
RT	451.68 (88.97)	486.51 (88.84)	421.14 (69.65)	447.68 (72.54)	514.51 (148.78)	531.21 (147.67)	444.56 (103.13)	482.07 (99.42)

For RT, significant main effects were identified for consistency and target face (*F*_(1, 42)_ = 27.39, *p* < .01, *η*^2^_p_ = .40, *BF*_1,0_ = 2322.06; *F*_(1, 42)_ = 49.22, *p* < .01, *η*^2^_p_ = .54, *BF*_1,0_ = 2.05×10^9^, respectively). Shorter RTs were identified in conditions of unconformity over conformity (*M* = 495.98, *SE* = 18.16 vs. *M* = 448.86, *SE* = 13.01), and following exposure to happy faces compared to neutral faces (*M* = 486.87, *SE* = 15.15, vs. *M* = 457.97, *SE* = 15.41). No main effect was found for game type (*F* < 2.00, *p* >.18). However, an interaction between game type × consistency × target face was identified (*F*_(1, 42)_ = 4.72, *p* = .035, *η*^2^_p_ = .10, *BF*_1,0_ = 3.06). A simple effect test was performed. For conformity conditions, slower search activity was observed for angry faces compared to happy faces in the violent video game group (*F*_(1, 42)_ = 16.21, *p* < .01, *η*^2^_p_ = .28); however, no difference was observed in the neutral video game group (*F*_(1, 42)_ = 3.72, *p* = .06 *η*^2^_p_ = .08. For the unconformity condition, a slower RT was observed for angry faces over happy faces in both groups (all *F* > 18.00, *p* < .01, *η*^2^_p_ > .31). No additional two-way interactions were identified for RT (all *F* < 1.00, *p* > .20).

### N2pc results

In order to examine the effects of video game exposure on the average N2pc amplitude, a 2 × 2 repeated-measures Bayes ANOVA was used to examine interactions between video game type (violent vs. neutral) and target face (angry vs. happy).

For task A, 42 participants were included the data analysis, as two participants demonstrated large artifacts (over 50% trail amplitudes exceeding ±100 μV). Our results indicated no main effect of target face, *F*_(1, 40)_ = 2.15, *p* = .15,*η*^2^_p_ = .051, *BF*_1,0_ = 1.56. In addition, no main effect of game type was found, *F*_(1,40)_ = .21, *p* = .649, *η*^2^_p_ = .001, *BF*_1,0_ = 2.94x10^-25^. There was no two-way interaction for game type and target face, *F*_(1,40)_ = .03, *p* = .876 *η*^2^_p_ = .001, *BF*_1,0_ = 2.94x10^-121^.

**Fig 4:**
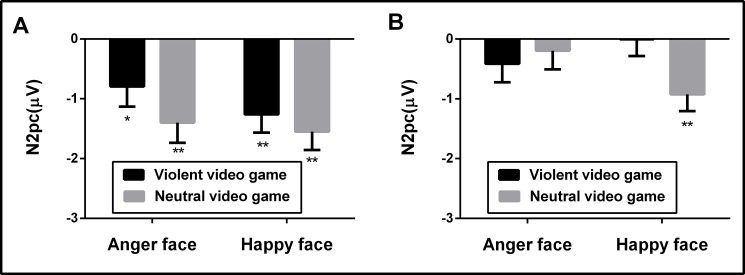
N2pc amplitudes. (A) Comparison of N2pc amplitudes in response to different facial affect images in task A. (B) Comparison of N2pc amplitude in response to different facial affect images in task B. Error bars indicate one standard error.

In order to detect the N2pc component in the two gaming groups, we compared the component measures with zero, and observed significant N2pc activity in the violent video game group in response to both angry (*t*_(20)_ = -4.36, *p* < .01, *d* = .97) and happy (*t*_(20)_ = -4.86, *p* < .01, *d* = 1.18) faces (**Fig 4A**). An N2pc component was also observed in the neutral video game group in response to both angry (*t*_(20)_ = -2.81, *p* = .011, *d* = .78) and happy (*t*_(20)_ = -4.40, *p* < .01, *d* = 1.14) faces.

Data from 41 participants were used for task B, since three participants demonstrated large artifacts (over 50% trail amplitudes exceeding ±100 μV). Our results indicated no main effect of target face, *F*_(1, 39)_ = .66, *p* = .421,*η*^2^_p_ = .017, *BF*_1,0_ = .892. In addition, no main effect of game type was found, *F*_(1,39)_ = .94, *p* = .339, *η*^2^_p_ = .025, *BF*_1,0_ = .295. There was a weak two-way interaction of game type and target face, *F*_(1,39)_ = 4.22, *p* = .046 *η*^2^_p_ = .097, *BF*_1,0_ = 2.56.

In order to detect the N2pc component in the two gaming groups, we compared the component measures with zero, and observed no N2pc activity in the violent video game group in response to either happy (*t*_(19)_ = -.01, *p* = .99, *d* = .01) or angry (*t*_(19)_ = -1.79, *p* = .09, *d* = .40) faces (**Fig 4B**). Despite this, N2pc activity was detected in the neutral video game group (*t*_(20)_ = -5.71, *p* < .01, *d* = 1.02), but not in response to angry faces (*t*_(20)_ = -.23, *p* = .82, *d* = .06). This therefore provides weak evidence for attentional capture having occurred in response to happy but not angry faces for the neutral video game group. In addition, regardless of the target face, the violent video game group exhibited difficulty in disengaging for task B.

## Discussion

In the present study, N2pc waveforms were used as a temporal marker of attentional selection processes during two emotional search tasks after short-term exposure to violent video games. No main effect was detected for game type and no interaction with target face was identified in task A, suggesting no difference in attentional facilitation between the violent and neutral video game groups. Furthermore, N2pc results suggested that attentional facilitation occurred in both groups.

In contrast to results observed in task A, task B revealed slower search activity for angry faces than happy faces during the conformity condition in the violent video game group. However, this slowing did not appear in the neutral video game group. This finding suggests that participants in the violent video game group exhibited lower attention when searching for angry faces. Furthermore, no N2pc activity was identified in response to angry faces for both violent and neutral video games. However, an N2pc component was observed in response to happy faces in the neutral, but not violent, video game group. N2pc activity reflects a mechanism involved in attentional selection and shifting in response to stimuli [25,27]. The present data suggest that, during task B, an increasing number of attentional resources were allocated to happy faces in subjects of the neutral video game group, but not to those in the violent video game group. This finding highlights the different effects of violent video game exposure on emotional search processes, and indicates potential underlying attentional mechanisms. Social information process theory [48] suggests that the attentional selection process is the first step required for coping with stimuli in individuals exposed to violence. Along with the abovementioned results, the present study demonstrated that individuals exposed to violence exhibited difficulty in disengaging from emotional faces, which could be a mechanism of attentional bias; however, this finding remains to be clarified.

### Attentional facilitation after violent video game exposure

The present study identified no effects of violent video game exposure on attentional facilitation, which did not support our predictions. Specifically, the N2pc results observed during task A suggested that attentional facilitation did not solely result from violent video game exposure, since both violent and neutral video games had the same effect on attentional bias in response to happy and angry faces, indicating that attention was focused on array switching. As previously mentioned, N2pc reflects the attentional selection process [25]; thus, our results reflected the same attentional process during the facilitation task. This finding is inconsistent with previous studies, which have indicated that angry faces are more quickly recognized after exposure to violent video games [25, 27]. There are three possible reasons for this observation. First, the tasks used for analysis in the present research differed from those employed in previous studies. For example, Kirsh et al. used a dynamic emotion identification task, which consisted of an emotional face that switched between neutral, happy, and angry faces. However, the present study used a static face array. Thus, previous studies predominantly focused on recognition processes, while the current study chose to investigate the attentional selection process. Another explanation for the discrepancy between our findings and the literature is that we focused on different information processes. In previous studies, a greater focus rested on the “happy face advantage”, which is reportedly reduced after exposure to violent video games. However, this study aimed to explore attentional resources allocated to both happy and angry faces after short-term exposure to violent video games, in which no “happy face advantage” or “angry face advantage” effect was observed. Finally, previous studies typically utilized different types of violent video games (e.g., *House of the Dead 2*) than what we employed in the present study (i.e., *Left 4 Dead*). This difference is crucial since violent games such as *House of the Dead 2* are more dread-based, while the game we employed was a shooting game, which was extremely bloody. However, despite inconsistencies with previous research, attentional facilitation was identified in response to both violent and neutral video games, and this finding is not consistent with the GAM theory that violent video games increase aggressive cognition (e.g., negative scripts).

### Difficulty in disengagement when searching for happy faces after violent video game exposure

N2pc responses during the attentional disengagement task reflect the top-down regulatory nature of attentional selection processes. A significant N2pc component was identified in response to happy faces following participant exposure to neutral but not violent video games. On the other hand, individuals exposed to violent video games experienced difficulty in disengagement when searching for happy faces; specifically, these participants took longer to distinguish happy faces from neutral faces. N2pc has been linked to attentional ability [25, 27], wherein a higher amplitude represents a greater distribution of attention. This is in agreement with the reduced N2pc amplitude we observed when participants searched for happy faces after violent video game exposure. N2pc has also been linked to selective attention in visual search tasks. While attentional facilitation and disengagement are different components of selective attention, the measure for each process is a distinguishing ability.

It is worth noting that no significant N2pc component was identified in response to angry faces in both the violent and neutral video game groups, which suggests that difficulty in disengagement was not unique to violent video game exposure. This finding was inconsistent with our predictions. Although research has been inconsistent, and marked by some significant flaws, there is some evidence that may indicate some action oriented games may influence social cue processing and attentional bias to threatening faces [13, 20], however the meaningfulness of these studies remains unclear. We suspect that the specificity of negative emotional faces (i.e., angry) might underlie this discrepancy; however, this will require further investigation to confirm.

The present findings are not consistent with previous action video game studies, which have reported that game exposure might improve cognitive shifting and attention [48]. However, the present study investigated only social information processing, rather than purely cognitive processing. Similarly, Bailey and West (2013) reported two effects of video game exposure: improved visuospatial attention and impaired emotional processing [19]. Together with their study, our results suggest that caution should be exercised when using action-based video games to modify visual processing, as this experience might exert unintended effects on emotional processing.

### Limitations and future research

Despite encouraging findings, the present study was subject to several limitations. First, while effort was made to control for difficulty, enjoyment, and other video game attributes, various aspects, such as competitiveness and arousal, were not matched. Indeed, a higher level of arousal was observed in almost all subjects following violent video game exposure when compared to those exposed to neutral video games. Therefore, future research must control for the abovementioned key variables as much as possible in order to help resolve debates regarding the effects of violent video games. Second, two typical types of emotional faces were employed in this study. Other studies have considered a wider range emotions, including disgust or sadness, which might produce different attention bias after exposure to violent video games. In particular, it remains unclear whether difficulties in disengagement from negative stimuli were due to desensitization resulting from violent video game exposure or difficulties in disengagement from negative stimuli. Future research should investigate this. Third, our participants were predominantly male. Although the majority of violent video game players are male, a balance of genders might be more suitable and could provide further insight on the effects of exposure to violent video games. In addition, individual variables, such as personality traits, were not considered in the present study. Previous research suggests that individuals with increased neuroticism, one of the “Big Five” personality traits, prefer to play violent video games and demonstrate attentional bias to negative stimuli [49, 50]. Further research should be undertaken to measure the interaction of this variable with violent video game playing.

With regard to debates into the effects of violent video games, the present study suffered from the above limitations, and was unable to provide direct evidence of the effects of violent video games on aggression. It might be that difficulties in disengagement result from violent video game exposure. However, caution should be exercised in the interpretation of our results, especially, with regard to this critical issue. Specifically, whether there exists a close association between violent video game exposure and aggressive behavior requires further research.

## Conclusions

While future investigation is needed, the present study identified the neural correlates of media violence on attentional and emotional face processing. To clarify the involvement of attentional component facilitation vs. difficulty in disengagement, we conducted two emotional search tasks after video game exposure (violent vs. neutral). Our results did not support the facilitation mechanism of violent video game exposure, indicating no significant difference in N2pc activity between the two game groups. However, reduced attentional capture was observed following violent video game exposure, supporting difficulty in disengagement as a potential attentional mechanism that might underlie the effects of violent video game exposure on affect processing. No N2pc component was observed in response to happy faces in individuals exposed to violent video games, but weak N2pc activity in response to happy faces was observed in individuals exposed to neutral video games. This provided only inconsistent support for the disengagement hypothesis, as we postulated that participants would exhibit difficulty disengaging only after violent video game exposure.

## Supporting information

S1 File The behavioral and ERP data for task A. (XLS)

S2 File The behavioral and ERP data for task B. (XLS)
